# Antithrombotic Therapy in the Prevention of Stroke

**DOI:** 10.3390/biomedicines9121906

**Published:** 2021-12-14

**Authors:** Shyamal Bir, Roger E. Kelley

**Affiliations:** Department of Neurology, Ochsner/LSU Health Sciences Center, Shreveport, LA 71130, USA; shyamal.bir@lsuhs.edu

**Keywords:** antiplatelet therapy, stroke prevention, TIA, minor stroke, anticoagulant therapy, atrial fibrillation, aspirin, clopidogrel, ticagrelor

## Abstract

Overview: Ischemic stroke is a leading cause of death and disability throughout the world. Antithrombotic therapy, which includes both antiplatelet and anticoagulant agents, is a primary medication of choice for the secondary prevention of stroke. However, the choices vary with the need to incorporate evolving, newer information into the clinical scenario. There is also the need to factor in co-morbid medical conditions as well as the cost ramifications for a particular patient as well as compliance with the regimen. Pertinent Updates: In the acute setting, dual antiplatelet therapy from three weeks to up to three months has become recognized as a reasonable approach for patients with either minor stroke or transient ischemic attack or those with symptoms associated with higher-grade intracranial stenosis. This approach is favored for non-cardioembolic stroke as a cardiogenic mechanism tends to be best managed with attention to the cardiac condition as well as anticoagulant therapy. Risk stratification for recurrent stroke is important in weighing potential risk versus benefits. For example, prolonged dual antiplatelet therapy, with a combination such as aspirin and clopidogrel or aspirin and ticagrelor, tends to have negation of the potential clinical benefit of stroke prevention, over time, by the enhanced bleeding risk. Anticoagulant choices are now impacted by newer agents, initially identified as novel oral anticoagulants (NOACs), which also became associated with “non-vitamin K” agents as they are no longer considered novel. Alternatively, they are now often identified as direct oral anticoagulants (DOACs). They tend to be viewed as superior or non-inferior to warfarin with the caveat that warfarin is still viewed as the agent of choice for stroke prevention in patients with mechanical heart valves. Conclusion: Based upon cumulative information from multiple clinical trials of secondary prevention of stroke, there is an increasing array of approaches in an effort to provide optimal management. Antithrombotic therapy, including in combination with anticoagulant therapy, continues to evolve with the general caveat that “one size does not fit all”. In view of this, we desire to provide an evidence-based approach for the prevention of stroke with antithrombotic agents.

## 1. Introduction

Stroke is a leading cause of death and neurological disability in the world. Stroke prevention is, therefore, a high priority in removing this substantial burden. The typical approach to acute ischemic stroke or transient ischemic attack (TIA) is to identify the mechanism of the ischemic insult as rapidly and accurately as possible. Typical mechanisms include small vessel (penetrating artery) ischemic stroke commonly referred to as lacunar-type infarct; large vessel athero-thrombotic stroke, which can result in large vessel occlusion (LVO); artery-to-artery embolic stroke; cardioembolic stroke; and embolic stroke of undetermined source (ESUS). Less common mechanisms include vascular dissection, vasculitis, and infectious vascular occlusive disease such as meningo-vascular syphilis. The mechanism can be compounded by underlying disorders such as a pro-thrombotic state or familial dyslipidemias, which can result in accelerated atherosclerosis.

Antithrombotic agents, either antiplatelet or anticoagulant, are a main stay of the prevention of ischemic stroke [1]. Evolving information has brought to the forefront the selection of dual antiplatelet therapy, such as aspirin and clopidogrel or aspirin and ticagrelor, in the acute setting of minor ischemic stroke or TIA if no clear contraindications are encountered. An embolic mechanism often calls for consideration of anticoagulant therapy, but this is impacted by the scenario such as high-grade carotid stenosis associated to artery-to-artery embolism, which generally is most effectively managed either with timely carotid endarterectomy or carotid angioplasty with stenting. A particularly challenging scenario can be the choice of antithrombotic regimen in ESUS [2].

## 2. Antithrombotic Agents

As mentioned previously, antithrombotic agents represent both antiplatelet agents and anticoagulant agents. Antiplatelet agents typically function by blocking the activation pathway of platelets while anticoagulants typically interfere with the coagulation cascade for clot formation. Antiplatelets are primarily used for the prevention of non-cardioembolic stroke while anticoagulants tend to be most efficacious for the prevention of cardioembolic stroke. Commonly used antiplatelet agents include aspirin; clopidogrel; dipyridamole; and a combination of low dose aspirin and clopidogrel, cilostazol, and ticagrelor. Antiplatelet agents either not commonly used or not of proven efficacy include ticlopidine, prasugrel, and eptifibatide. Anticoagulant choices include warfarin, rivaroxaban, apixaban, edoxaban, and dabigatran.

## 3. Antiplatelet Therapy for the Prevention of Stroke

### 3.1. Aspirin

Aspirin is the most commonly used antiplatelet agent used for stroke prevention. This reflects its established efficacy, low cost, relative safety, and ease of use with generally limited drug–drug interaction. It is available without a prescription, which is beneficial in terms of cost and availability. However, this can also translate into a variable pattern of administration with medication reconciliation efforts. A not uncommon scenario, in the acute ischemic stroke setting, is trying to determine whether the patient is actually taking the medication, and how regularly, as the use of such an agent may not be recorded on the medication list of prescribed medications and its as often as necessary use, for pain, may cause it to be confused with acetaminophen or a non-steroidal anti-inflammatory agent when trying to obtain this information in the acute setting. 

In terms of mechanism of action, aspirin administration results in the irreversible blockage of both cyclo-oxygenase (COX) enzymes. COX 1 blockage causes inhibition of platelet thromboxane A_2_ (TXA_2_) [3]. The half-life of aspirin is 2 to 3 h [4,5]. The more common side effects of aspirin include dyspepsia and gastrointestinal bleeding. It is uncommon to see easy bruising reflective of its antiplatelet effect, and this may provide reassurance that it is being taken regularly. Fortunately, intracranial hemorrhage is an uncommon complication [4,5,6]. Aspirin is contraindicated if there is a reported history of significant allergic reaction. A history of asthma, nasal polyps, and rhinitis can be associated with an enhanced risk of bronchospasm, urticaria, and angioedema although significant allergic reactions are not commonly observed.

A combined analysis of 40,000 patients from CAST (Chinese Acute Stroke Trial) and IST (International Stroke Trial) demonstrated that early initiation of aspirin, at 160 to 300 mg a day, reduces the risk of stroke recurrence compared to control [7]. In the CAPRIE trial [8], comparison of clopidogrel at 75 mg daily versus aspirin at 325 mg daily showed no significant difference in the recurrence rate of stroke, MI, and vascular death in the 19,183 ischemic stroke patients reported. Therefore, aspirin is typically viewed as first-line antiplatelet therapy for stroke prevention in non-cardioembolic ischemic stroke [7]. In meta-analysis data, aspirin, in a dosage range of 81 to 325 mg a day, was associated with an approximately 13% relative reduction of recurrence of stroke [8]. There has been no reported efficacy difference between 81 and 325 mg a day for protection against vascular events including stroke [9].

Aspirin is of limited efficacy for stroke prevention in embolic stroke with a cardiogenic source. The Stroke Prevention in Atrial Fibrillation (SPAF) I [10] study assessed aspirin, at 325 mg a day, compared to placebo and to warfarin in patients without prior stroke who had non-valvular atrial fibrillation (NVAF). There was a reduction in ischemic stroke with aspirin (3.6%/year) compared to placebo (6.3%/year) as well as in combined ischemic stroke and death (7.9%/year versus 11.8%/year). Warfarin was clearly superior to aspirin in stroke prevention in this study and in subsequent studies of NVAF. Bleeding risk was comparable in all groups. As per the American Heart Association (AHA) guidelines, aspirin is viewed as a reasonable alternative to anticoagulant therapy for those not eligible for an anticoagulant because of safety concerns as well as in case of refusal or lack of compliance with the anticoagulant [11].

A not uncommon scenario is the so-called “aspirin failure”, which identifies a patient presenting with ischemic stroke or TIA while presumably compliant with the prescribed aspirin. This often results in the patient being switched to an alternative antiplatelet agent, such as clopidogrel, and it is increasingly common to initiate dual antiplatelet therapy (DAPT) in the acute period. Aspirin resistance is an important concern in the vascular neurology community. One study reported that the prevalence of ASA resistance is 25% [12]. Another study reported that diabetes mellitus and high LDL cholesterol independently cause aspirin resistance by the upregulation of isoprostanes and subsequent activation of arachidonic acid. This finding was supported by high levels of 11-dehydrothromboxane B2, a breakdown product of thormboxane A_2,_ in the urine of these subjects [13]. Additional contributing factors may include concurrent use of a proton-pump inhibitor reported to be associated with higher platelet aggregation and higher serum levels of thromboxane B_2_ [14]. Additionally of note, enteric-coated aspirin formulations were found to be associated with a reduced inhibition of thromboxane A_2_ related to reduced absorption, with the neutral pH, of the small intestine [15].

### 3.2. Clopidogrel

Clopidogrel is commonly used for vascular prophylaxis because of its established efficacy, reasonable side effect profile, and reasonable cost associated with its availability in generic form. It is a prodrug, which is metabolized to its active form by carboxylesterase-1. There is resultant irreversible platelet inhibition through binding to PGY_12_-ADP receptors on the platelet’s surface. This prevention of ADP binding to the PGY_12_ receptors results in activation of the glycoprotein GPII b/IIIa complex with the resultant inhibition of platelet aggregation [16].

The CAPRIE trial [17] compared aspirin, at 325 mg a day, to clopidogrel, at 75 mg a day, in 19,185 patients with either recent ischemic stroke, recent MI, or peripheral arterial disease. The relative risk reduction for a composite of either ischemic stroke, MI, or vascular death was 8.7% (*p* = 0.043) favoring clopidogrel over aspirin. Thus, the benefit was not necessarily to the degree that clopidogrel supplanted aspirin as the first choice for antiplatelet therapy, but it did identify this agent as an option for “aspirin failures”.

Like aspirin, clopidogrel enhances bleeding risk, and its enhanced efficacy in platelet inhibition warrants concern for invasive procedures, which could lead to a clinically significant bleeding complication. This translates into holding of the clopidogrel for 5 to 7 days, at least in the non-emergent clinical setting, prior to surgical procedures [18], and this includes procedures, such as the placement of a feeding gastrostomy and lumbar puncture, which are not uncommonly encountered in the neurological realm.

The peak of platelet inhibition of clopidogrel usually occurs in 3 to 5 days after initial administration of the standard 75 mg a day dose while a loading dose of 300 to 600 mg can show an effect at six hours [19,20]. Clopidogrel efficacy is predicated upon genetic-based responsiveness to its mechanism of action. The loss-of-function CYP2C19*2 allele is associated with decreased activation of clopidogrel, and its antiplatelet effect, as well as worse cardiovascular event outcome [21].

## 4. Combined Aspirin and Clopidogrel Therapy

With different mechanisms of action, the combination of aspirin and clopidogrel was theorized to be more effective than either agent alone in vascular event protection as long as the safety profile was similar to monotherapy. This was addressed, in a preliminary fashion by Meyer et al. [22]. Three trials assessed this combination with non-supportive results. MATCH [23] looked at clopidogrel 75 mg a day plus aspirin at 75 mg a day versus clopidogrel alone in 7599 patients with recent ischemic stroke or TIA plus at least one additional vascular risk factor. The relative risk reduction for the primary endpoint was 6.4% ((*p* = 0.244), while any potential benefit was negated by the worrisome bleeding risk of longer-term DAPT. The CHARISMA trial looked at clopidogrel at 75 mg a day plus aspirin at 75 to 162 mg a day versus clopidogrel versus aspirin at 75 to 162 mg/day in patients with vascular disease including stroke and TIA (4300 out of 15,603 patients) [24]. There was no significant protection seen in one group over the other in terms vascular outcomes. SP3 [25] assessed 3020 patients with lacunar stroke treated with either clopidogrel 75 mg/day plus aspirin at 75 to 162 mg/day or aspirin alone at 75 to 162 mg/day within six months of presentation. There was no benefit seen with DAPT, but the risk for major hemorrhage with DAPT was significant (*p* = 0.004). Thus, longer-term DAPT therapy was abandoned until further studies reported potential benefit for shorter timeframes of therapy in the acute setting.

The ACTIVE investigators assessed DAPT compared to warfarin, with an INR of 2 to 3, in NVAF [26], with no benefit observed for secondary stroke prevention. In a follow-up study, these investigators looked at clopidogrel at 75 mg/day plus aspirin at 75 to 100 mg/day compared to aspirin alone in NVAF [27]. A reduced major vascular event rate was reported, over a 3.6 year follow-up, on average, but the bleeding event rate tended to negate overall benefit.

The SAMMPRIS trial, published in 2011, looked at angioplasty and stenting in symptomatic higher-graded intracranial stenosis (≥70%) with medical therapy, consisting of aspirin 325 mg a day and clopidogrel 75 mg a day compared to medical therapy in combination with angioplasty and stenting [28]. The “aggressive” medical therapy alone was not only superior to the intervention group at 90 days but also when compared to historical controls. This led to the potential application of DAPT for a limited course in higher-risk patients.

The CHANCE study [29] assessed 5170 patients treated with an antiplatelet regimen within 24 h of presentation with either a minor ischemic stroke (NIHSS ≤ 3) or higher-risk TIA (ABCD^2^ score ≥4). A clopidogrel load of 300 mg followed by 75 mg a day plus aspirin at 75 mg a day was compared to aspirin 75 mg a day monotherapy. There was a reported significant reduction in recurrent stroke without an associated significant risk of hemorrhage in the DAPT group at 90 days follow up. In the POINT study [30], 4881 patients with minor stroke or higher-risk TIA were treated with either clopidogrel 600 mg loading dose followed by 75 mg a day and aspirin 50 to 325 mg a day or aspirin alone within 12 h of presentation. There was significant reduction in major ischemic events, with DAPT, but also a significant risk for major bleeding compared to aspirin alone. This was at 90 days follow up, with the bleeding risk becoming more of a concern over time.

A number of meta-analyses have been performed that support short-term DAPT use in selected patients and when given within 24 h of onset of symptoms. Combined data from the CHANCE and POINT trials led to the recommendation that DAPT be confined to the first 21 days [31]. Hao et al. [32] reported a 20 in 1000 patient reduction in subsequent stroke with a 2 per thousand increased risk of moderate to severe bleeding. Similar conclusions were echoed by Ye et al. [33], while Hillman et al. cited a 0.2% risk of major hemorrhage with aspirin alone compared to 0.9% with aspirin and clopidogrel [34].

In an extension of their CHANCE study, Wang et al. [35] reported reduced efficacy for stroke prevention in the subgroup of patients who were carriers of CYP2C19 loss-of-function alleles [35]. Genetic polymorphism of the *ABCD1* involved in the intestinal absorption of clopidogrel may also impact potential efficacy of this antiplatelet agent [36].

## 5. Dipyridamole plus Aspirin

Dipyridamole inhibits the platelet cyclic adenosine monophosphate (cAMP) phosphodiesterase and breakdown of adenosine as well as the potentiation of PGI activity and synthesis. In prevention of stroke, monotherapy with dipyridamole is rarely use because of its unclear efficacy [3,5]. The combination of low-dose aspirin with higher-dose, extended-release dipyridamole came to the forefront based on the European Stroke Prevention Study 2 (ESPS 2) [37]. The dosing was 25 mg of aspirin combined with 200 mg of the dipyridamole formulation twice a day.

The ESPS 2 reported that a composite of stroke or death was reduced by 13.2% (*p* = 0.016) with aspirin alone, by 15.4% for higher-dose dipyridamole (*p* = 0.015), and by 24.4% for the combination ((*p* < 0.001). Similar findings were reported in ESPRIT [38]. In this study, 2739 patients were entered within six months of an ischemic stroke or TIA, compared to 6602 in the ESPS 2 with subjects entered within three months. The absolute risk reduction in vascular events was 3% in both studies with no significant difference in the major bleeding rate between aspirin alone and the combination. Despite the improvement, the adoption of low-dose aspirin with higher-dose, extended-release dipyridamole was tempered by the not uncommonly observed significant headache.

In the Prevention Regimen for Effectively Avoiding Second Strokes (PRoFESS) trial, aspirin/dipyridamole was compared to clopidogrel monotherapy in 20,332 patients with an ischemic stroke within 90 days of randomization [39]. There was no statistical difference in reduction of stroke, MI, or vascular death with somewhat higher concern for major bleeding in the aspirin/dipyridamole group as well as a higher frequency of significant headache. In the Triple Antiplatelets for Reducing Dependency after Ischemic Stroke (TARDIS) trial, triple therapy with aspirin/dipyridamole and clopidogrel was compared to monotherapy [40]. There was no reduction in recurrent stroke, but there was an enhanced risk of bleeding with triple therapy.

## 6. Ticagrelor

This is a newer antiplatelet agent with the mechanism of action involved with the selective inhibition of the binding of adenosine phosphate to its platelet receptor (P2Y12), without being metabolized, with resultant platelet aggregation inhibition [3,5]. As for other antiplatelet agents, bleeding risk and allergic reaction are the two major safety concerns.

In the SOCRATES trial [41], ticagrelor, with a 180 mg loading dose followed by 90 mg twice a day was compared to aspirin, with a loading dose of 300 mg followed by 100 mg a day, in patients with mild to moderate ischemic stroke and TIA. The hazard ratio (HR) favored ticagrelor for ischemic stroke prevention, HR = 0.87, *p* = 0.046; with a trend in terms of reduction of a composite of stroke, MI, and death, HR = 0.89, *p* = 0.07; while the major bleeding risk was increased but not statistically significant, HR = 0.83, *p* = 0.45. In the THALES trial [42], ticagrelor plus aspirin was compared to aspirin alone following mild to moderate ischemic non-cardioembolic stroke (NIHSS ≤ 5) or high-risk TIA (ABCD^2^ score ≥ 6). The HR was 0.83 favoring the combination with *p* = 0.02 for the primary outcome of stroke or death within 30 days. Ischemic stroke within 30 days was seen in 5.0% of those assigned to combination therapy compared to 6.3% in the aspirin alone group (HR = 0.79, *p* = 0.004), but the incidence of disability was not significantly different. In addition, the risk of severe bleeding was higher with the combination (0.5%) compared to aspirin alone (0.1%), *p* = 0.001. Thus, ticagrelor is now available, used typically in combination with aspirin, for the prevention of stroke with a finite window of up to 30 days according to this study.

## 7. Cilostazol

Cilostazol is an approved antiplatelet agent for peripheral arterial disease as well as thrombotic complications of coronary angioplasty when used in combination with aspirin or clopidogrel [43]. Its mechanism of action involves selective inhibition of phosphodiesterase 3, which increases the activation of intracellular cAMP and protein kinase A, resulting in the inhibition of platelet aggregation. In addition, cilostazol may prevent recurrent ischemic stroke by improving endothelial function [44]. Cilostazol has been the focus of at least 18 randomized trials of secondary stroke prevention as outlined in a meta-analysis by Tan et al. [45]. The dosing was typically 100 mg twice a day with or without aspirin. Of note, cilostazol was reported to compare favorably with both aspirin and clopidogrel for secondary stroke prevention as well as safety. However, improvement in neither in mortality reduction nor functional outcome was observed in this meta-analysis.

## 8. Primary Prevention of Stroke

The primary prevention of stroke with antiplatelet therapy has been somewhat arbitrary based upon conflicting reports. Naturally, the approach is typically with a relatively safe and cost-effective medication, following the principle of “do no harm”. Typically, there is differentiation from “higher-risk” patients without history of TIA or ischemic stroke but with co-morbid predisposing factors such as longstanding hypertension, diabetes mellitus, or strong family history of ischemic events. Such patients are often empirically placed on aspirin for primary prevention, while those with documented coronary artery or peripheral arterial disease tend to be routinely placed on an antiplatelet regimen. In subjects without cardiovascular disease or stroke, there is a reported lower absolute risk of ischemic stroke based upon cumulative information from 10 randomized clinical trials [46]. The absolute risk was reported as 1.27% with aspirin compared to 1.48% without aspirin, HR = 0.81, with the number that needed to be treated to prevent one event being 241. Furthermore, the absolute risk of major bleeding in 12 randomized controlled trials reveals a higher risk in the aspirin group 23.1% vs. 16.4% with an absolute risk increase of 0.47%, and the number needed to cause an adverse event at 210. Thus, there is very limited support for aspirin as a primary stroke prevention medication in lower-risk patients.

## 9. Summary of Antiplatelet Therapy for Secondary Stroke Prevention

There are now several choices in terms of selection of antiplatelet therapy for secondary stroke prevention, with aspirin tending to remain the first choice as long as there are no contraindications. Table 1 summarizes the agents and their mechanism of action. Patients with coexistent coronary artery disease, particularly those with stents, might be better managed with DAPT for at least a finite period of time. Clopidogrel or combined aspirin/dipyridamole is particularly indicated for “aspirin failures”. Ticagrelor, especially in combination with aspirin, provides additional efficacy but with added concern about bleeding complications. Cilostazol is now also a potential component of the antiplatelet armamentarium for secondary stroke prevention especially if alternative approaches are not successful.

## 10. Anticoagulants in the Prevention of Stroke

The WARSS trial outlined the limitation of anticoagulant therapy with warfarin in the secondary prevention of non-cardioembolic stroke [47]. No difference between aspirin and warfarin was observed. Atrial fibrillation (AF) can occur in isolation or in association with congenital and acquired valve-related disease. Non-valvular AF (NVAF) increases the risk of ischemic stroke 5-fold and risk is increased up to 17-fold in the patients with AF and mitral stenosis [48]. A number of studies [10,49,50,51] have demonstrated the superiority of warfarin over aspirin in the prevention of stroke with (NVAF), and this protection extends an even greater justification for anticoagulant therapy in valvular AF. Anticoagulants are generally recommended for secondary prevention of stroke in patients with cardioembolic stroke. For example, the incidence of embolic events in valvular AF is eight times higher in patients without oral anticoagulant therapy than in those with anticoagulant therapy [52].

## 11. Presently Available Anticoagulants for Stroke Prevention

These anticoagulant agents include warfarin, rivaroxaban, apixaban, edoxaban, and dabigatran.

### 11.1. Warfarin

Warfarin inhibits the activity or synthesis of vitamin-K-dependent clotting factors including II, VII, IX, and X. Warfarin has been used for a number of years for protection against cardioembolic stroke and for protection against stroke associated with hypercoagulable conditions. It has been replaced by DOACs for many patients with NVAF but not for valvular-associated AF based upon results of the RE-ALIGN [53]. Persistent advantages of warfarin use, despite the inconvenience associated with drug–drug and food interactions with variable effect on the therapeutic dosing, are the ability to determine compliance by the determination of a therapeutic prothrombin time (PT) and international normalized ration (INR); adjustment of dosing for higher-risk clinical conditions; and the reversibility of the anticoagulant effect, when indicated for bleeding, which only recently became available for the DOACs. In addition, the ready availability of a PT/INR measurement in the emergency setting helps in determining potential eligibility for intravenous tissue plasminogen activator (TPA) for acute ischemic stroke, while patients on DOACs are routinely contraindicated for TPA unless it is well documented that they have not recently been receiving the medication.

### 11.2. Direct Oral Anticoagulants (DOACs)

Direct oral anticoagulants include rivaroxaban, apixaban, and edoxaban (which directly inhibit activated factor X) and dabigatran (which is a direct inhibitor of thrombin). Studies have shown that these DOACs have similar or superior efficacy in preventing thromboembolic strokes as well as a reduced or similar intracranial bleeding risk compared to warfarin. The major benefits of these DOACs over warfarin are [1] fixed dosing regimen, [2] no requirement of monitoring of PT/INR, [3] fewer drug–drug interactions, and [4] rapid and predictable onset of action [4,5,39]. Contraindication of DOACS are severe renal impairment and the required adjustment of dosing for moderate impairment or advanced age. Not unexpectedly, the major side effect concern of DOACs is excessive bleeding [5]. Available reversal agents for dabigatran and factor Xa inhibitors are idarucizumab (a monoclonal antibody) and andexanet alfa (engineered version of factor Xa), respectively [4].

The RE-LY (Randomized Evaluation of Long-Term Anticoagulation Therapy) study with dabigatran 110 or 150 mg twice daily compared to warfarin (INR 2-3) [53] showed that dabigatran is superior to warfarin, at the 150 mg twice a day dose, and non-inferior to warfarin at the 110 mg twice a day dose, in the reduction of stroke and systemic embolism in the patients with NVAF. Dabigatran had a similar rate to warfarin for major hemorrhage.

The AVERROES trials [54,55] and the ARISTOTLE-AF study [56] demonstrated that apixaban was superior to warfarin in reducing the risk of recurrence of stroke and systemic embolism without increasing the risk of major bleeding. However, among patients with prior stroke or TIA, there was no significant difference in reduction of stroke and systemic embolism between apixaban (2.5%) and warfarin (3.2%). The ROCKET-AF study showed that rivaroxaban 20 mg daily compared to warfarin (INR 2–3) showed that rivaroxaban is non-inferior to warfarin in reduction of stroke and systemic embolism in patients with non-valvular atrial fibrillation. In addition, the risk of major bleeding was comparatively less in the rivaroxaban group [57]. The ENGAGE-AF and -TIMI 48 studies demonstrated that edoxaban was non-inferior to warfarin in terms of reduction of recurrent strokes, systemic thromboembolism, major bleeding, and death [58,59].

The RIVER clinical trial in the patients with AF and bioprosthetic mitral valve showed that rivaroxaban was non-inferior to warfarin, with a slight tendency to decrease the prevention of stroke, death, and major bleeding at 12 months [60]. However, in the RE-ALIGN study of patients with AF and mechanical heart valves [61], the occurrence of stroke was 5% with dabigatran and 0% with warfarin. Major bleeding was also higher in the dabigatran group (dabigatran 4% vs. warfarin 2%). Presently, warfarin remains the agent of choice for protection against cardioembolism in patients with AF and mechanical heart valves as well as in those subjects with clinically significant mitral stenosis.

The timing for starting an anticoagulant is particularly important for chronic anticoagulant therapy in the patients with stroke and NVAF or VAF. Current guidelines recommend waiting for 4–14 days in a patient with hemorrhagic transformation or in patients with malignant ischemic stroke with AF. The presently available anticoagulant choices are summarized in Table 2.

### 11.3. Combination Therapy of DOAC and Aspirin in Stable Atherosclerotic Vascular Disease without Atrial Fibrillation

The COMPASS trial was a study in 27,395 patients with stable atherosclerotic vascular disease and reported that 2.5 mg rivaroxaban twice a day plus 100 mg of aspirin daily showed reduction of stroke, MI, and cardiovascular death compared to aspirin alone. Rivaroxaban 5 mg twice a day showed similar results to 2.5 mg rivaroxaban twice a day plus 100 mg of ASA daily but was associated with a higher rate of major bleeding [62]. Of note, this study excluded the patients with prior history of intracerebral hemorrhage, prior stroke within one month, and lacunar stroke. The major trials on anticoagulants for secondary prevention are depicted in Table 3.

### 11.4. Embolic Stroke of Undetermined Source (ESUS)

Embolic stroke of undetermined source is an emerging area of clinical interest and research with particular emphasis on risk versus potential benefit. The RE-SPECT ESUS trial showed dabigatran is not superior to aspirin in secondary stroke prevention in such patients and demonstrated more non-major bleeding than aspirin [63]. The NAVIGATE ESUS trial, with rivaroxaban in comparison with aspirin, reported an unacceptable bleeding risk with rivaroxaban, and the study was prematurely terminated [64]. Thus, at the present time, at least until further study information is available, antiplatelet therapy remains the avenue of choice for ESUS [65].

### 11.5. Antithrombotic after Receiving Mechanical Thrombectomy in Acute Stage of Stroke

Periprocedural antiplatelet therapy and continuation of the therapy after mechanical thrombectomy (MT) shows better functional outcomes [66]. The current guideline recommends starting antiplatelets after 24 h of MT if repeat CTH shows no bleed. If MT confirms complete reperfusion, patient should be on ASA 81 mg daily or clopidogrel 75 mg daily depending on history of antithrombotic intake for secondary prevention of stroke. However, in case of no or incomplete perfusion, patient can be placed on dual antiplatelets with ASA and clopidogrel for 3 months followed by monotherapy. If patient has history of atrial fibrillation (a-fib) or newly diagnosed a-fib, oral anticoagulant such as apixaban or rivaroxaban should be the choice of treatment after MT with no bleed or malignant MCA stroke in repeat CT head. If any patient has hemorrhagic transformation from ischemic stroke in repeat CT head, antithrombotic or anticoagulant should be resumed after 4–14 days of stroke onset depending on the size of hemorrhage [67]. In case of concurrent internal carotid artery occlusion and when the patient needs to undergo carotid stenting along with mechanical thrombectomy, the choice of treatment should be periprocedural antiplatelets, and life-long continuation of that [68].

## 12. Future Direction

The successful intra-arterial use of tPA showed better outcome in acute stroke from proximal arterial occlusion. Intra-arterial tPA is being used in some centers now, and most probably it will be universalized in the near future as the benefit of IV tPA is limited in acute stroke from large proximal arterial occlusion. The use of IV tPA along with mechanical thrombectomy (MT) showed no extra benefit compared to MT alone. Therefore, use of IV tPA in the patients who are undergoing mechanical thrombectomy for arterial occlusion may be obsolete in future. Newer thrombolytics aiming toward fibrin specificity with rapid onset of action, shorter half-life, better safety, and efficacy should be established [69]. Tenecteplase would be an alternative for IV tPA. Moreover, judicial use of neuroprotectants including nerinetide (an eicosapeptide) or magnesium would be beneficial in acute stroke. Treatment with ASA and ticagrelor has been shown better neurological outcome in minor stroke or high-risk TIA, and the above combination would be an alternative to ASA and clopidogrel as clopidogrel showed resistance in certain populations and required platelet function assays. Lastly, clopidogrel and cilostazol would also be an alternative combined therapy for high-risk non-cardioembolic stroke.

## 13. Summary

This review highlights the increasing options we have available for our patients for relatively safe and effective stroke prevention with antithrombotic therapy. Aspirin tends to be the first choice with DAPT being a consideration in the shorter term for non-cardioembolic stroke. The subset of patients who experience recurrent cerebral events, despite the more standard antiplatelet regimen choices, may be eligible for cilostazol or combination of low-dose anticoagulant with aspirin. The DOACs are preferable to warfarin for stroke prevention in AF in most patients; however, those who are limited in their access to these newer agents, or have contraindications, may be better suited for warfarin, particularly those with significant coexistent valvular heart disease or significant renal impairment.

## Figures and Tables

**Table 1:** Antiplatelet choices with mechanism of action.

Agent	Mechanism of Cation
Aspirin	Irreversible blockage of both cyclo-oxygenase (COX) enzymes. COX 1 blockag cause inhibition of platelet thromboxane A_2_ (TXA)
Clopidogrel	A prodrug which is metabolized to its active form by carboxylesterase 1. There is resultant irreversible platelet inhibition through binding to PGY_12_-AD receptors on the platelets surface. This prevention of ADP binding to the PGY_12_ receptors results in activation of the glycoprotein GPII b/IIIa complex with resultant inhibition of platelet aggregation.
Dipyridamole	Inhibits the platelet cyclic adenosine monophosphate (cAMP) phosphodiesterase and breakdown of adenosine as well as potentiation of PGI activity and synthesis.
Ticagrelor	selective inhibition of the binding of adenosine phosphate to its platelet receptor (P2Y12), without being metabolized, with resultant platelet aggregation inhibition
Cilostazol	selective inhibition of phosphodiesterase 3 which increases activation of intracellular cAMP and protein kinase A, which results in inhibition of platelet aggregation

**Table 2:** Anticoagulant choices, dosing, and comparative efficacy in non-valvular atrial fibrillation.

Agent	Dosing	Comparitive Efficacy to Warfarin
Digabatran	150 mg twice daily (75mg twice daily incase of renal impairment	Superior
Apixaban	5 mg twice a day (2.5 mg twice a day for age ≥ 80, BMI <60 kg or serum creatinine >1.5	Superior
Rivaroxaban	20 mg a day (15 mg a day with renal impairment	Non-inferior
Edoxaban	60 mg a day (do not use fro CrCL greater than 95ml/min because of an increased risk of ischemic stroke compared with warfarin in a NVAF trial)	Non-inferior
Warfarin	Targated INR of 2 to 3	Not applicable

**Table 3:** Major trials on antithrombotics.

Trial Name and References	# of Patients	Treatment Arms	Primary End Points	Results and *p* Value
CAST and ISC trial [7]	40,000	Aspirin 160 to 300 mg daily vs. placebo (control)	Recurrence of ischemic stroke within 30 days	Recurrence of ischemic stroke. Aspirin 1.6% vs. placebo 2.3%, *p* < 0.000001
SPAF; Stroke Prevention in Atrial Fibrillation [10]	1330	Aspirin 325 mg, warfarin, and placebo in patients with atrial fibrillation	Recurrence of ischemic stroke and death	Recurrence of ischemic stroke: aspirin 42% (*p* = 0.02), warfarin 67% (*p* = 0.01). Death: aspirin 32% (*p* = 0.02) and warfarin 58% (*p* = 0.01)
CAPRIE trial [17]	19,185	Aspirin 325 mg vs. clopidogrel 75 mg daily	Recurrence of ischemic stroke	Reduction of recurrence of ischemic stroke was 8.7% in favor of the clopidogrel group (*p* = 0.043)
MATCH trial [23]	7599	DAPT, aspirin 75 mg, and clopidogrel 75 mg vs. clopidogrel 75 mg daily	Recurrence of ischemic stroke	Reduction of recurrence of ischemic stroke: 6.4% (*p* = 0.244), no benefit of using DAPT
POINT trial [30]	4881	Aspirin 50 to 325 mg and clopidogrel 75 daily (first load with 600 mg) vs. aspirin 50 to 325 mg daily for 21 days of onset in minor ischemic stroke or higher-risk TIA	Recurrence of ischemic stroke	Recurrence of ischemic stroke is 5% in combined group vs. 6.5% in aspirin monotherapy group, *p* = 0.02
CHANCE [29]	5170	Aspirin 75 mg and clopidogrel 75 daily (first load with 300 mg) vs. placebo and aspirin 75 mg daily for 21 days of onset in minor ischemic stroke or higher-risk TIA	Recurrence of ischemic stroke	Recurrence of ischemic stroke is 8.2% in combined group vs. 11.75% in aspirin monotherapy group, *p* < 0.001
SAMMPRIS trial [28]	451	Aspirin 325 mg and clopidogrel 75 mg daily vs. medical therapy in combination with angioplasty and stenting in a patient with 70–99% intracranial stenosis for 90 days	Recurrence of ischemic stroke and death	Recurrence of ischemic stroke and death: medical management with angioplasty and stentin, 14.7% vs. medical management only 5.8%, (*p* = 0.002)
European Stroke Prevention Study 2 (ESPS 2) 37	6602	Aspirin 75 mg and dipyridamole 200 mg twice daily vs. aspirin 75 mg or dipyridamole 200 mg daily	Recurrence of ischemic stroke and death	Reduced the goal by 13.2% (*p* = 0.016) with aspirin alone, and 15.4% by higher dose of dipyridamole (*p* = 0.015), and 24.4% by the combination (*p* < 0.001)
SOCRATES trial [41]	13,199	Ticagrelor 90 mg twice daily (first loaded with 180 mg) vs. aspirin 100 mg daily (first loaded with 300 mg) in mild to moderate ischemic stroke and TIA for 90 days	Recurrence of ischemic stroke	Recurrence of ischemic stroke, ticagrelor 6.7% vs. aspirin 7.5%, *p* = 0.07. No benefit was noted.
THALES trial [42]	11,016	Ticagrelor 90 mg twice daily (first loaded with 180 mg) plus aspirin was compared to aspirin 75 to 100 mg daily (first loaded with 300 to 325 mg) in mild to moderate ischemic stroke and TIA	Recurrence of ischemic stroke	Recurrence of ischemic stroke; 5.0% for combination therapy compared to 6.3% for aspirin, *p* = 0.004
RE-ALIGN study [61]	252	Dabigatran vs. warfarin in patients with mechanical heart valve	Recurrence of ischemic stroke and major bleeding	Recurrent strokes: 5% with dabigatran and 0% with warfarin, and major bleeding 4% vs. 2%, respectively.
RE-LY study [53]	18,113	Warfarin vs. dabigatran 110 or 150 mg daily in patients with atrial fibrillation and stroke	Recurrence of ischemic stroke or systemic embolization	Warfarin 1.69% vs. dabigatran 1.53% (non-inferior to warfarin), *p* < 0.001 vs. dabigatran 150 mg, 1.11% (superior to warfarin), *p* < 0.001
AVERROES trials [55]	18,201	Apixaban 5 mg twice a day vs. warfarin in atrial fibrillation	Recurrence of ischemic stroke or systemic embolization	Apixaban, 1.27% vs. warfarin, 1.67%, *p* < 0.01 (superiority)
ROCKET-AF study [57]	14,264	Rivaroxaban 20 mg daily vs. warfarin in non-valvular atrial fibrillation	Recurrence of ischemic stroke or systemic embolization	Recurrence of stroke was 1.7% with rivaroxaban vs. 2.2% with warfarin, *p* < 0.001 (rivaroxaban is non-inferior to warfarin)
COMPASS trial [62]	27,395	Rivaroxaban 2.5 mg twice a day plus aspirin 75 mg and aspirin 75 mg daily in non-atrial fibrillation stroke	Recurrence of ischemic stroke, MI, and cardiovascular death	Primary outcome; stroke; 4.1% in combined group vs. 5.4% in aspirin alone group, *p* < 0.001, and death; 3.4% vs. 4.1%, *p* = 0.00254; however, major bleeding was 3.1% vs. 1.9%, respectively, *p* = 0.001

## Data Availability

Not applicable.
